# Malaria transmission dynamics surrounding the first nationwide long-lasting insecticidal net distribution in Papua New Guinea

**DOI:** 10.1186/s12936-015-1067-7

**Published:** 2016-01-12

**Authors:** Lisa J. Reimer, Edward K. Thomsen, Gussy Koimbu, John B. Keven, Ivo Mueller, Peter M. Siba, James W. Kazura, Manuel W. Hetzel, Peter A. Zimmerman

**Affiliations:** Case Western Reserve University, Cleveland, OH USA; Papua New Guinea Institute of Medical Research, Goroka, Papua New Guinea; Liverpool School of Tropical Medicine, Liverpool, UK; Michigan State University, East Lansing, MI USA; Walter and Eliza Hall Institute, Parkville, VIC Australia; Swiss Tropical and Public Health Institute, Basel, Switzerland; University of Basel, Basel, Switzerland

**Keywords:** *Anopheles punctulatus*, *Anopheles farauti*, *Anopheles kolienses*, Malaria, Papua New Guinea, Bed nets, LLIN

## Abstract

**Background:**

The major malaria vectors of Papua New Guinea exhibit heterogeneities in distribution, biting behaviour and malaria infection levels. Long-lasting, insecticide-treated nets (LLINs), distributed as part of the National Malaria Control Programme, are the primary intervention targeting malaria transmission. This study evaluated the impact of LLINs on anopheline density, species composition, feeding behaviour, and malaria transmission.

**Methods:**

Mosquitoes were collected by human landing catch in 11 villages from East Sepik Province and Madang Province. Mosquitoes were collected for 3 years (1 year before distribution and 2 years after), and assayed to determine mosquito species and *Plasmodium* spp. infection prevalence. The influence of weather conditions and the presence of people and animals on biting density was determined. Determinants of biting density and sporozoite prevalence were analysed by generalized estimating equations (GEE).

**Results:**

Mosquito biting rates and entomological inoculation rates decreased significantly after the distribution. *Plasmodium falciparum* and *P. vivax* sporozoite prevalence decreased in year 2, but increased in year 3, suggesting the likelihood of resurgence in transmission if low biting rates are not maintained. An earlier shift in the median biting time of *Anopheles punctulatus* and *An. farauti s.s*. was observed. However, this was not accompanied by an increase in the proportion of infective bites occurring before 2200 hours. A change in species composition was observed, which resulted in dominance of *An. punctulatus* in Dreikikir region, but a decrease in *An. punctulatus* in the Madang region. When controlling for village and study year, *An. farauti s.s.*, *An. koliensis* and *An. punctulatus* were equally likely to carry *P. vivax* sporozoites. However, *An. punctulatus* was significantly more likely than *An. farauti s.s.* (OR 0.14; p = 0.007) or *An. koliensis* (OR 0.27; p < 0.001) to carry *P. falciparum* sporozoites.

**Conclusions:**

LLINs had a significant impact on malaria transmission, despite exophagic and crepuscular feeding behaviours of dominant vectors. Changes in species composition and feeding behaviour were observed, but their epidemiological significance will depend on their durability over time.

**Electronic supplementary material:**

The online version of this article (doi:10.1186/s12936-015-1067-7) contains supplementary material, which is available to authorized users.

## Background

Malaria transmission in Papua New Guinea (PNG) is highly variable across environmentally diverse zones, ranging from intense perennial transmission in the northern coastal lowlands to seasonal moderate transmission in the southern coast and unstable transmission at higher altitudes [1]. A recent survey reported weighted malaria parasite prevalence of 12 % nationwide, with substantial heterogeneity, ranging from 0 to 49.7 % [2]. Four malaria species are endemic to PNG (*Plasmodium falciparum*, *P. ivax*, *P. ovale*, and *P. malariae*) with the majority of infections caused by *P. falciparum* and *P. vivax* [3, 4]. The major malaria and filariasis vectors in PNG are members of the *Anopheles punctulatus* group. This group comprises 13 species, each exhibiting different degrees of exophily and anthropophily and different habitat preferences [5–8]. The five major malaria vectors in this group, due to their widespread distribution and high abundance, include *An. punctulatus*, *An. farauti s.s*., *An. koliensis*, *An. hinesorum*, and *An. farauti* 4 [5]. In addition to the *Anopheles punctulatus* group, *An. bancroftii*, *An. longirostris*, *An. karwari*, and *An. subpictus* have been incriminated as malaria vectors.

Prior to the development of more sensitive molecular diagnostics in the 1990s [9–11], identification was restricted to the Punctulatus clade (now known to include *An. punctulatus*, *An. farauti* 4 and *An.* sp.* nr punctulatus*), Farauti clade (now known to include *An. farauti s.s., An. hinesorum, An. torresiensis, An. farauti* 5*, An. farauti* 6, and *An. farauti* 7) and *An. koliensis*. Morphological identification, based on proboscis scale patterns and the presence of a sector spot on the costal wing vein, was often unreliable in distinguishing *An. koliensis* due to variable scaling patterns, even within isofemale lines [12–15].

Although restricted by morphological identification, early studies highlight heterogeneities in habitat preference, seasonality, human blood index, and transmission potential among members of this complex. Differences were observed in the spatial distribution of species among and within villages, larval habitats and vegetation. *An. farauti s.s.* is primarily found in coastal villages with a high tolerance for breeding in brackish water. *An. farauti s.s.* and *An. hinesorum* are more commonly found in natural breeding sites, such as ground pools, while *An. punctulatus* is commonly found in areas disturbed by human activity [14, 16]. *An. punctulatus* is abundant in the hills and *An. koliensis* has a patchy distribution in lowland inland areas [17], and both exploit different larval habitats [18]. Abundance is correlated with recent rainfall, with *An. koliensis* showing greater temporal variability than *An. punctulatus* and *An. farauti s.l.* Peak outdoor biting times vary with the majority of *An. farauti s.l.* biting in the early evening and *An. koliensis* and *An. punctulatus* biting in the late night and early hours of the morning [13].

Pilot projects in PNG in the 1950s demonstrated the likelihood that DDT could successfully control malaria given the proper resources. It was not until the 1970s, through the support of United Nations Development Programme, that the DDT indoor residual spraying campaign was scaled up to cover over 50 % of the population [19]. In Madang, DDT spraying was ineffective against *An. farauti s.l.,* but very effective against *An. punctulatus* and moderately effective against *An. koliensis* [17]. In the Solomon Islands, DDT residual spraying impacted members of the complex differently [20, 21] with a stronger impact on *An. punctulatus* and *An. koliensis* than *An. farauti s.s.*

Long-lasting, insecticide-treated nets (LLINs) can be a powerful tool in reducing malaria-associated morbidity, particularly when high community coverage is achieved [22]. In PNG, where the vector population is 100 % susceptible to pyrethroids [23] LLINs remain effective for up to 5 years of in-home use [24], and are an attractive choice for malaria control. However, the tendency of the vector population to spend the majority of time outdoors [17] may render them less susceptible to indoor vector control due to low biological coverage. Furthermore, home-based interventions may have an unequal impact in areas with multiple vector species exhibiting a range of exophily and anthropophily.

The PNG Malaria Control Programme, supported by the Global Fund to Fight AIDS, Tuberculosis and Malaria, launched a countrywide, free, LLIN distribution between 2005 and 2009. PermaNet 2.0 LLINs, treated with 55 mg/cu m deltamethrin and manufactured by Vestergaard Frandsen, were distributed at a target ratio of one net per 2.5 household members. Independent household surveys reported 68.7 % of households surveyed in the Momase region (Morobe, Madang and Sepik Provinces included in this study) owned at least one LLIN (with 47 % sleeping underneath a LLIN the night before), while 95 % of households had at least one net of any type (with 74 % sleeping underneath the night before) [25]. In addition, the malaria control programme introduced rapid diagnostic tests and artemisinin-based combination therapy in 2012 [26].

The purpose of this study was to determine the impact of the LLIN distribution on mosquito abundance, species composition, peak biting times, and malaria transmission in three geographical regions of PNG with multiple, sympatric vector species.

## Methods

### Study villages

**Fig. 1:**
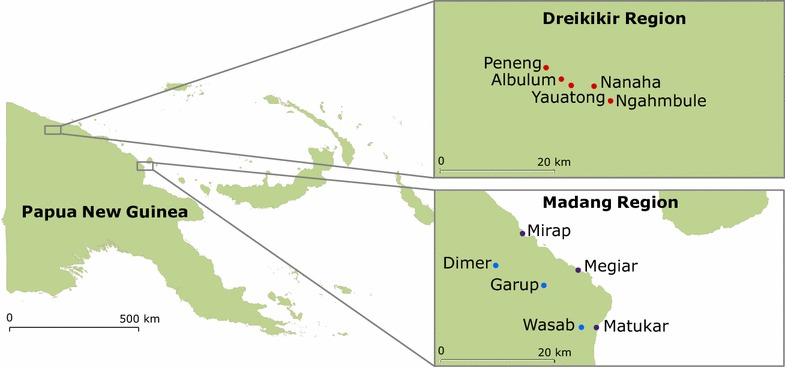
Map of study villages. Madang coastal (*purple*), Madang inland (*blue*) and Dreikikir (*red*) field sites

Eleven villages, covering three geographic regions, were included in the study (Fig. 1). Coastal villages are characterized by coconut plantations and swamps while the inland foothills (Madang and Dreikikir) are characterized by thick vegetation. Houses are typically built on stilts from bamboo or sago palm with a large open veranda. July, August and September experience lower rainfall but there is no pronounced dry season. Climate, topography and larval sites have been described elsewhere [14].

### Experimental design

Mosquitoes were collected by the outdoor human landing catch method for 1 year prior to and 1 year following the nationwide LLIN distribution. Four representative villages (Matukar, Dimer, Nanaha, Yauatong) were chosen for further surveillance throughout year 3. Collectors were trained to aspirate host-seeking mosquitoes before biting in order to minimize exposure to malaria. Pairs of collectors worked in teams, with one individual collecting all mosquitoes landing on exposed legs from 1800 to 2400 hours and the second individual collecting from 24.00 to 06.00. The collectors rotated from the early shift to the late shift on subsequent nights. Mosquitoes were stored in cups according to the date, location and hour of collection. Following morphological identification [27, 28], mosquitoes were stored dry on silica gel and returned to the laboratory for mosquito species confirmation and enzyme-linked immunosorbent assay to detect *Plasmodium* spp. circumsporozoite protein. In the Madang villages, additional data were recorded hourly by each collector, including: the number of animals and number of additional people present in the hamlet, as well as a qualitative record of wind, rainfall and cloud cover (none, light, moderate or heavy).

### Collection effort

In both inland and coastal regions of Madang, the first year of mosquito collections began in August 2008 until LLINs were distributed in July and August 2009. Year 2 collections began after LLIN distribution through September 2010, and year 3 collections concluded in November 2011. In the Dreikikir region, the first year of collections began in September 2008 until LLINs were distributed in late August 2009. Year 2 collections continued until July 2010 and year 3 collections concluded in July 2011. Mosquitoes were collected monthly in every village for the first 2 years. In the third year, collections occurred only in select villages with bimonthly collections in Dimer and Matukar and quarterly collections in Nanaha and Yauatong. Details of collection effort per year are shown in Additional file 1: Table S1.

### Molecular diagnostics

Mosquitoes that were morphologically identified as members of the *An. punctulatus* group were confirmed to species by PCR-restriction fragment length polymorphism of the ITS2 region (4) using either an individual leg or extracted DNA (QIAGEN, Maryland, USA). Lysates from whole mosquitoes were screened for *P. falciparum*, *P. vivax* 210 and *P. vivax* 247 circumsporozoite proteins by enzyme-linked immunosorbent assay [29].

### Rainfall data

Rainfall data were collected by the PNG National Weather Service from Madang airport, 37–52 km from the Madang study villages. Although variations are to be expected between inland and coastal villages, regional rainfall records demonstrate typical seasonality and a lack of aberrant rainfall during the collection period.

## Ethical approval

This study was approved by the institutional review boards at University Hospitals Case Medical Center in Cleveland, Ohio, USA and the Institute of Medical Research and Medical Research Advisory Council of PNG.

## Data analysis

Generalized estimating equations (GEE) were used to identify the determinants of mosquito abundance and infection prevalence. When the dependent variable was binary (sporozoite prevalence), a binomial distribution with a log link function was used. For count data (mosquito abundance), a Poisson distribution with log link function was used. In both cases, an exchangeable working correlation matrix was assumed. For sporozoite prevalence, separate models were constructed with either *P. falciparum* or *P. vivax* positivity as the dependent variable. Mosquito species (only *An. punctulatus* complex members were included) and year of the study (years 1, 2, 3) were covariates and village was the subject variable. For mosquito abundance, LLINs (presence or absence), rain in mm the previous month, the number of people and animals present outdoors within the compound, and the qualitative variables of rain, wind and cloud cover were included as covariates. Subject variables were village and collector, and within-subject variables were the date and hour of collection.

Species composition before and after the distribution are presented for the four intensively surveyed villages. Due to a significant association between *P. falciparum* infection and *An. punctulatus*, the proportion of this species of the total catch between year 1 and years 2 and 3 was compared by Fisher’s exact test.

Median biting times were calculated based on the entire catch of a given species per village and year. Not all collected species could be PCR-confirmed, therefore only villages that showed 95 % concordance between morphological and molecular identification in a given year are presented, and the data include all morphologically identified species. Mann–Whitney U tests were performed to determine if the medians between years were the same.

Mean man-biting rates were calculated for each village and compared between years 1 and 2 or 1 and 3 (*t* test with Bonferroni correction for multiple comparisons). Entomological inoculation rates, a measure of the number of infective bites per person per year, were calculated based on the average total number of bites per person per year and the sporozoite prevalence in mosquitoes.

## Results

The distribution of LLINs had a significant effect on anopheline biting density (p < 0.001, Table 1). In addition to the presence of LLINs, wind and rain at the time of collection were negatively associated with anopheline biting density (p < 0.001 and p = 0.046, respectively), as was rainfall from the previous month (p = 0.002). Cloud cover at the time of collection was positively associated with biting density (p = 0.003) although this relationship was limited to light cloud cover only. The number of people and animals present in the hamlet during the collection hour did not influence biting density (p = 0.56 and p = 0.80, respectively).

**Table 1:** The effect of environmental variables on hourly catch of anopheline mosquitoes

Variable	Category	p value	Odds ratio	95 % CI
Wind^a^	None		1.00	
	Light	0.263	0.87	[0.69, 1.11]
	Moderate	<0.001	0.50	[0.39, 0.66]
	Heavy	<0.001	0.37	[0.27, 0.52]
Cloud^a^	None		1.00	
	Light	0.001	1.40	[1.14, 1.72]
	Moderate	0.245	0.83	[0.60, 1.14]
	Heavy	0.890	0.99	[0.83, 1.18]
Rain^a^	None		1.00	
	Light	0.563	1.06	[0.87, 1.30]
	Moderate	0.120	0.69	[0.44, 1.10]
	Heavy	0.129	0.76	[0.54, 1.08]
LLINs^b^	Absent		1.00	
	Present	<0.001	0.38	[0.29, 0.48]
People^c^		0.555		
Animal^c^		0.800		
Rain_lag^d^		0.002		

^a^Categorical variables refer to the conditions during the hour of collection

^b^Categorical variable referring to whether LLINs had been distributed

^c^Continuous variables refer to the number of alternative hosts present during the hour of collection

^d^Continuous variable referring to the amount of rainfall in the previous month

**Fig. 2:**
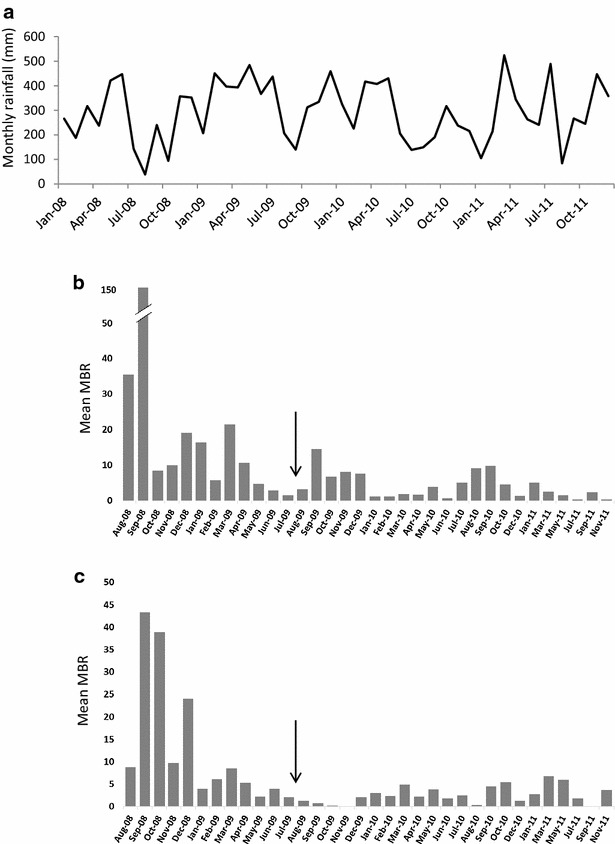
Madang region monthly rainfall and biting rates. Madang airport monthly rainfall (**a**) and mean nightly man biting rates in (**b**) coastal Madang (n = 5093), (**c**) inland Madang (n = 2804). The *arrows* represent LLIN distribution dates in the community. Each village had a similar sampling effort between years 1 and 2 (August 2008–July 2010). However, in year 3 (August 2010–November 2011), only Matukar and Dimer were sampled

Mean monthly man biting rates for the Madang regions are shown in Fig. 2. In both Madang sites, biting densities peaked in September 2008 and generally declined until LLINs were distributed in August 2009. This peak corresponds with low rainfall in August following heavier rains in June (Fig. 2a). Similar patterns in rainfall were observed during all three years; however, after LLINs were distributed biting densities remained low (Fig. 2b, c).

**Fig. 3:**
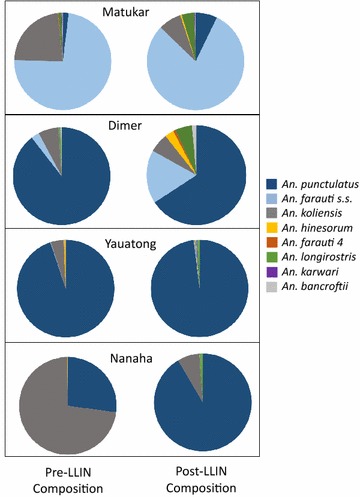
Species composition before and after LLIN distribution. PCR confirmed anopheline species composition for Matukar (n = 1182 pre-LLIN, n = 430 post-LLIN), Dimer (n = 1417 pre-LLIN, n = 272 post-LLIN), Yauatong (n = 722 pre-LLIN, n = 324 post-LLIN), Nanaha (n = 738 pre-LLIN, n = 83 post-LLIN)

*Anopheles farauti s.s*. was the dominant species collected in the coastal villages and *An. punctulatus* was dominant in inland Madang and Dreikikir (Fig. 3). *An. koliensis* was dominant in Nanaha, but only in year 1 (Fig. 3). In addition to members of the *An. punctulatus* group, *An. bancroftii*, *An. longirostris* and *An. karwari* were collected at lower densities. An increase in the proportion of *An. punctulatus* and a decrease in the proportion of *An. farauti s.s.* were observed in both representative Madang villages (Matukar: p = 0.0001, Dimer: p = 0.0001), while an increase in the proportion of *An. punctulatus* and a decrease in the proportion of *An. koliensis* were observed at both representative Dreikikir villages (Yauatong: p = 0.007, Nanaha: p = 0.0001).

**Fig. 4:**
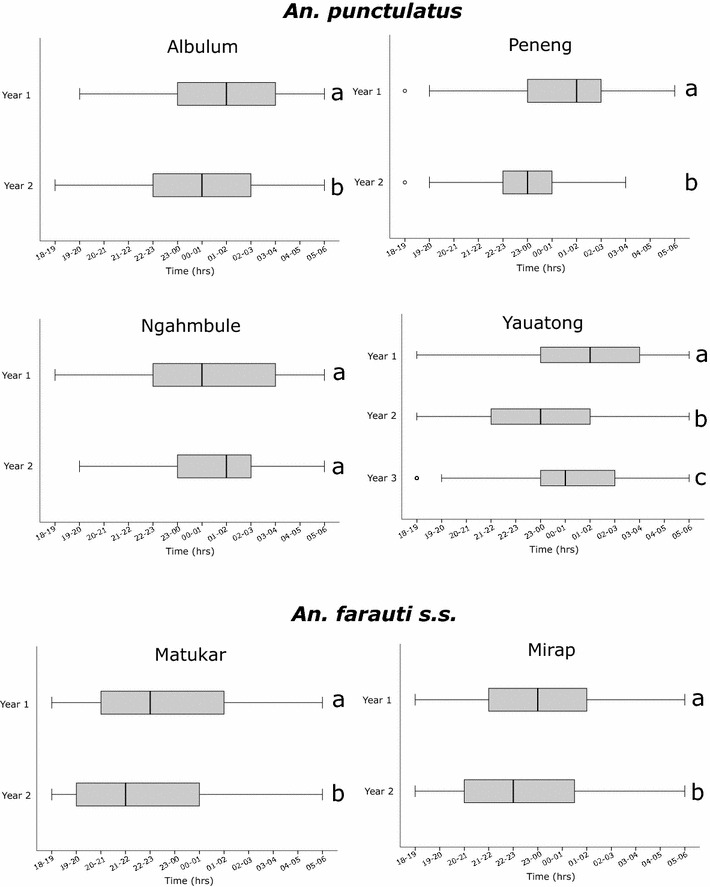
Biting time before and after LLIN distribution.* Boxes* indicate first to third quartile and median hours of biting activity.* Whiskers* represent fifth to 95th percentiles. Year 1 was before LLIN distribution and years 2 and 3 were after.* Boxes* carrying the same letter were not statistically different (Bonferroni adjusted alpha = 0.007)) when comparing median biting times using Mann–Whitney U tests. Albulum year 1 n = 874, year 2 n = 383; Peneng year 1 n = 715, year 2 = 103; Ngahmbule year 1 n = 596, year 2 = 100; Yauatong year 1 n = 2818, year 2 n = 672, year 3 n = 464; Matukar year 1 n = 2187, year 2 n = 187; Mirap year 1 n = 1191, year 2 n = 328

*Anopheles farauti s.s.* had a tendency for earlier biting compared to *An. punctulatus* and *An. koliensis* with 38, 16 and 15 % biting before 22.00, respectively. Significant shifts were observed in median biting time after the LLIN distribution for *An. punctulatus* and *An. farauti s.s.* (Fig. 4), with the exception of *An. punctulatus* from Ngahmbule village. Despite this shift, there was no statistically significant difference in the proportion of infective bites occurring before 22.00 pre-LLIN (42.9 %) compared to after LLINs (35 %, Fisher’s exact p = 0.56).

**Fig. 5:**
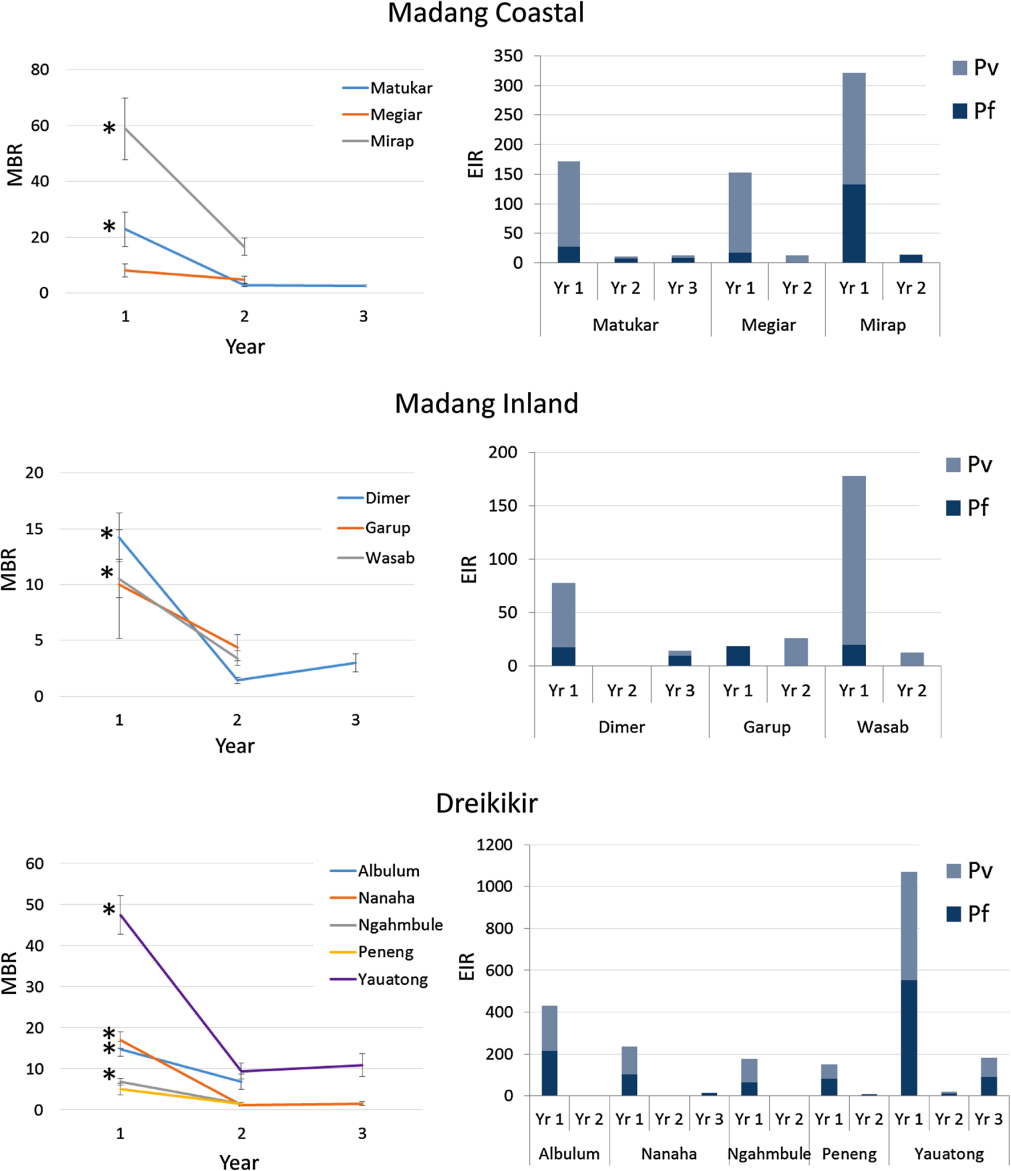
Man biting rate and entomological inoculation rate. Panels on the left show mean nightly biting rate (± SEM) in villages from each region. *Asterisk* indicates villages which experienced a significant reduction between years 1 (pre-LLIN) and 2 or 3 (post LLIN) (Bonferonni adjusted alpha = 0.003). Panels on the right show entomological inoculation rate (infective bites/person/year) with *P. falciparum* and *P. vivax* in year 1 (pre-LLIN), 2 and 3 (post-LLIN) for each region

Individual village level analyses showed a significant reduction in mean annual man biting rates in eight of the 11 villages following the LLIN distribution (Fig. 5; p < 0.003). The three remaining villages had the lowest pre-intervention biting rates per region and they exhibited reductions that were non-significant (Megiar p = 0.11, Garup p = 0.13, Peneng p = 0.009). A reduction in annual entomological inoculation rate (Fig. 5) was observed in all villages except Garup.

**Fig. 6:**
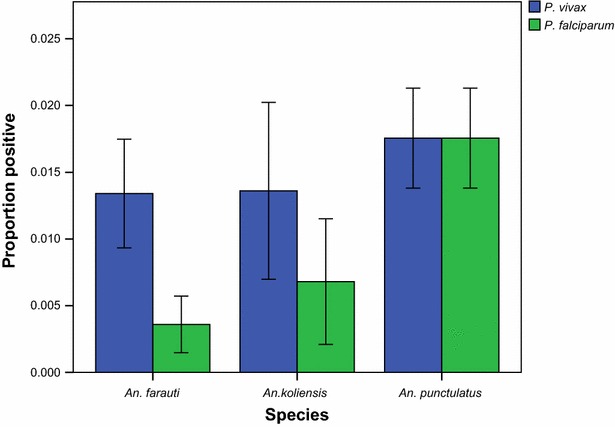
*Plasmodium* spp. sporozoite prevalence in dominant anophelines

GEE analysis indicated that, when controlling for village and mosquito species, the chance of a mosquito carrying sporozoites in the second year was significantly lower than the pre-intervention period for both *P. falciparum* (OR 0.12; 95 % CI 0.03, 0.51; p = 0.004) and *P. vivax* (OR 0.17; 95 % CI 0.08, 0.36; p < 0.001). In the third year, the odds of sporozoite positivity rebounded for both *P. vivax* (OR 0.49; 95 % CI 0.27, 0.87; p = 0.015) and *P. falciparum* (OR 0.74; 95 % CI 0.44, 1.25; p = 0.261), although the probability of carrying *P. vivax* sporozoites was still less than in the pre-intervention period (Additional file 2: Table S2). When controlling for village and study year, *An. farauti s.s.*, *An. koliensis* and *An. punctulatus* were equally likely to carry *P. vivax* sporozoites. However, *An. punctulatus* was significantly more likely than *An. farauti s.s.* (OR 0.14; 95 % CI 0.03, 0.58; p = 0.007) or *An. koliensis* (OR 0.27; 95 % CI 0.13, 0.57; p < 0.001) to carry *P. falciparum* sporozoites (Fig. 6).

## Discussion

Mosquito populations have been extensively studied in Madang and East Sepik Provinces, where *P. falciparum* and *P. vivax* are both highly endemic. In Dreikikir, pre-LLIN sporozoite rates and man biting rates were similar to those observed in the late 1950s [30]. In Madang Province, sporozoite rates are similar to previous studies [13, 31] but observed man biting rates were lower than historically reported [31, 32].

Overall, man biting rates dropped significantly in the year following LLIN distribution and remained low in representative villages through the third year. Even though members of the *An. punctulatus* group do not exhibit preferences for endophagy [17], LLINs may still have a large communal impact if LLIN coverage and usage is high [33]. LLIN usage in study sites was higher than regionally reported [25]. A survey 3 months following the distribution in Dreikikir revealed that 79 % had slept under a LLIN the previous night [34]. A separate survey conducted in Madang study sites 3 years after the distribution revealed that 79 % had slept under a LLIN in Mirap and 54 % had slept under a LLIN in Wasab (JBK, unpublished). A separate study has shown that individual LLIN use and high community LLIN coverage were independently and strongly associated with reduced odds of malaria infection in PNG [2].

Although biting rates decreased, a trend towards earlier biting was observed in *An. punctulatus* and *An. farautis.s*. Changes in biting behaviour were previously reported in *An. farauti* and *An. koliensis*, in a nearby village, following distribution of insecticide-treated nets in 1985 [35]. In PNG, this shift could be indicative of behavioural flexibility or the impact of LLINs on population age structure; it has been observed that nulliparous females bite earlier than parous females [36]. The former scenario has been suggested in an *An. farauti s.l.* population in the Solomon Islands following a DDT spraying campaign [20]. Additional behavioural and genetic studies could elucidate whether selection is occurring in populations in this study. In the latter scenario, early biting patterns would not be accompanied by increased exposure even if biting densities returned to pre-control levels. No increase in the proportion of infective bites occurring before 22.00 in this study was observed, although sample sizes were limited. Continued surveillance can determine if the observed shifts in biting times are epidemiologically significant, and whether they will limit the protective effect of LLINs.

Differences in species composition were observed after LLIN distribution. However, there was not a consistent trend across villages towards species dominance. For example, in the Madang villages, *An. farauti s.s.* increased in proportion over *An. punctulatus* while in Dreikikir, *An. punctulatus* increased in proportion over *An. koliensis.* It is likely that some species will be more resilient to this intervention than others, and changes in species composition will depend on behaviour and physiology of the entire population. The observed pattern may be attributed to a greater impact of LLINs on the later biting species in the community. The observed increase in the proportion of *An. punctulatus* could be epidemiologically significant given the close association between this species and *P. falciparum* transmission. Further work is needed to determine whether these changes in species composition are durable, and whether they are associated with malaria prevalence.

While LLINs were very effective in reducing man biting rate and entomological inoculation rate in the three regions, sporozoite prevalence did not remain low. The observed increase in sporozoite prevalence between years 2 and 3 may be attributable to a low sample size, a challenge in post-intervention evaluations. However, it is clear that residual malaria transmission is still occurring, and that any rebound in mosquito density will be accompanied by an increase in transmission intensity. In both Madang regions, *P. falciparum* sporozoite rate was higher in year 3 than in year 1. This result is surprising because vector control is expected to have a greater impact on *P. falciparum* than *P. vivax*, due in part to the longer extrinsic incubation period for *P. falciparum* and the role of dormant hypnozoites in *P. vivax* transmission. These higher infection rates in year 3 may be influenced by low mosquito numbers and further work to determine long-term trends is needed.

## Conclusions

This study has demonstrated a strong community impact of LLINs on exophagic and early biting vector populations. Success may be attributable to high LLIN ownership as well as a portion of the vector population that continues host seeking during the time when people are indoors. Individual malaria exposure will be affected by house construction, personal bed net use and other human behavioural patterns. Although LLINs had a clear impact on vector populations, the impact on sporozoite prevalence did not extend to year 3. Given the plasticity of mosquito behaviour and natural fluctuations in vector densities, continued surveillance is needed to determine changes in the effectiveness of LLINs.

## Additional file

10.1186/s12936-015-1067-7 Annual collection effort and total anophelines from each village.
10.1186/s12936-015-1067-7 Prevalence of *Plasmodium* spp. in wild anophelines.
