# Supplementary material for: Malaria transmission dynamics surrounding the first nationwide long-lasting insecticidal net distribution in Papua New Guinea
Source: Malar J. 2016 Jan 12;15:25. doi: 10.1186/s12936-015-1067-7 (PMC4709896; doi:10.1186/s12936-015-1067-7)
Supplement: Supplementary file 1 — 10.1186/s12936-015-1067-7 Annual collection effort and total anophelines from each village. [file 12936_2015_1067_MOESM1_ESM.docx]

Table S1. Annual collection effort (number of person-nights) and total anophelines collected in each village

|  |  |  | |  | | |  | | |  |  |  |  |  |  |  |
| --- | --- | --- | --- | --- | --- | --- | --- | --- | --- | --- | --- | --- | --- | --- | --- | --- |
|  |  | Year 1 | | Year 2 | | | Year 3 | | |  |  |  |  |  |  |  |
| Region | Village | Effort | Total | Effort | Total | Effort | | Total |  |  |  |  |  |  |  |  |
| Coastal | Matukar | 110 | 2512 | 120 | 334 | 86 | | 211 |  |  |  |  |  |  |  |  |
|  | Megiar | 22 | 177 | 28 | 133 |  | |  |  |  |  |  |  |  |  |  |
|  | Mirap | 22 | 1295 | 26 | 431 |  | |  |  |  |  |  |  |  |  |  |
| Inland | Dimer | 120 | 1709 | 120 | 171 | 76 | | 227 |  |  |  |  |  |  |  |  |
|  | Garup | 24 | 241 | 28 | 122 |  | |  |  |  |  |  |  |  |  |  |
|  | Wasab | 22 | 232 | 30 | 102 |  | |  |  |  |  |  |  |  |  |  |
| Dreikikir | Albulum | 60 | 890 | 68 | 467 |  | |  |  |  |  |  |  |  |  |  |
|  | Nanaha | 144 | 2445 | 80 | 91 | 46 | | 71 |  |  |  |  |  |  |  |  |
|  | Ngahmbule | 88 | 603 | 80 | 123 |  | |  |  |  |  |  |  |  |  |  |
|  | Peneng | 144 | 736 | 80 | 119 |  | |  |  |  |  |  |  |  |  |  |
|  | Yauatong | 60 | 2848 | 80 | 757 | 46 | | 500 |  |  |  |  |  |  |  |  |
|  |  |  |  |  |  |  | |  |  |  |  |  |  |  |  |  |
