# Supplementary material for: Malaria transmission dynamics surrounding the first nationwide long-lasting insecticidal net distribution in Papua New Guinea
Source: Malar J. 2016 Jan 12;15:25. doi: 10.1186/s12936-015-1067-7 (PMC4709896; doi:10.1186/s12936-015-1067-7)
Supplement: Supplementary file 2 — 10.1186/s12936-015-1067-7 Prevalence of Plasmodium spp. in wild anophelines. [file 12936_2015_1067_MOESM2_ESM.docx]

Table S2. Prevalence of *Plasmodium falciparum* and *P. vivax* in wild mosquitoes collected before (Year 1) and

After (Years 2-3) the national LLIN distribution

|  |  | Year 1 | | | Year 2 | | | Year 3 | | |
| --- | --- | --- | --- | --- | --- | --- | --- | --- | --- | --- |
| Region | Species | Pf prev (n) | Pv prev (n) | N | Pf prev (n) | Pv prev (n) | N | Pf prev (n) | Pv prev (n) | N |
| Madang Coastal | *An. farauti s.s.* | 0.0048 (10) | 0.0162 (34) | 2105 | 0.0027 (2) | 0.0027 (2) | 741 | 0.0128 (2) | 0.0064 (1) | 156 |
|  | *An. koliensis* | 0.0061 (2) | 0.0061 (2) | 329 | 0.0233 (1) | - | 43 | - | - | 23 |
|  | *An. punctulatus* | - | 0.0377 (2) | 53 | - | - | 27 | - | - | 28 |
|  | *An. longirostris* | - | 0.0625 (1) | 16 | - | - | 21 | - | - | 0 |
|  | *An. farauti 4* | - | - | 2 | - | - | 0 | - | - | 0 |
|  | *An. hinesorum* | - | - | 0 | - | - | 2 | - | - | 1 |
|  | *An. bancrofti* | - | - | 1 | - | - | 1 | - | - | 0 |
|  | *An. karwari* | - | - | 3 | - | - | 0 | - | - | 1 |
|  | *An. subpictus* | - | - | 15 | - | - | 0 | - | - | 0 |
|  | ***TOTAL*** | **0.0048** | **0.0155** | **2524** | **0.0036** | **0.0024** | **835** | **0.0096** | **0.0048** | **209** |
| Madang Inland | *An. punctulatus* | 0.0040 (6) | 0.0079 (12) | 1511 | - | 0.0042 (1) | 239 | 0.0115 (2) | 0.0057 (1) | 174 |
|  | *An. koliensis* | 0.0047 (1) | 0.0095 (2) | 211 | - | 0.025 (1) | 40 | - | - | 5 |
|  | *An. farauti* | - | 0.0291 (3) | 103 | - | 0.0208 (1) | 48 | - | - | 32 |
|  | *An. longirostris* | - | 0.1765 (6) | 34 | - | - | 60 | - | - | 4 |
|  | *An. bancrofti* | - | 0.5 (2) | 4 | - | - | 1 | - | - | 3 |
|  | *An. hinesorum* | - | - | 0 | - | - | 4 | - | - | 7 |
|  | *An. farauti 4* | - | - | 1 | - | - | 3 | - | - | 0 |
|  | ***TOTAL*** | **0.0038** | **0.0134** | **1864** | **-** | **0.0076** | **395** | **0.0089** | **0.0044** | **225** |
| Dreikikir | *An. punctulatus* | 0.0354 (71) | 0.0339 (68) | 2006 | 0.0025 (2) | 0.0013 (1) | 791 | 0.0241 (4) | 0.0181 (3) | 166 |
|  | *An. koliensis* | 0.0068 (4) | 0.0186 (11) | 590 | - | - | 11 | - | - | 3 |
|  | *An. hinesorum* | - | 0.1 (1) | 10 | - | - | 2 | - | - | 0 |
|  | *An. longirostris* | - | - | 0 | - | - | 2 | - | - | 2 |
|  | *An. farauti 4* | - | - | 0 | - | - | 3 | - | - | 0 |
|  | ***TOTAL*** | **0.0288** | **0.0307** | **2606** | **0.0025** | **0.0012** | **809** | **0.0234** | **0.0175** | **171** |
